# The Use of Lentinan for Treating Gastric Cancer

**DOI:** 10.2174/1871520611313050002

**Published:** 2013-06

**Authors:** Kenji Ina, Takae Kataoka, Takafumi Ando

**Affiliations:** 1 Department of Medical Oncology, Nagoya Memorial Hospital, Nagoya 468-8520, Japan; 2 Department of Clinical Oncology, Nagoya Memorial Hospital, Nagoya 468-8520, Japan; 3 Department of Gastroenterology, Nagoya Graduate School of Medicine, Nagoya 466-8550

**Keywords:** Gastric cancer, β-glucan, Lentinan.

## Abstract

Natural compounds containing fungal β-glucans have been used to improve general health for thousands of years in China and Japan. Lentinan, the backbone of β-(1, 3)-glucan with β-(1, 6) branches, is one of the active ingredients purified from *Shiitake* mushrooms and has been approved as a biological response modifier for the treatment of gastric cancer in Japan. Despite recent advances in chemotherapeutic agents, unresectable or recurrent gastric cancer remains an incurable disease, with survival rates being far from satisfactory. Recent clinical studies have shown that chemo-immunotherapy using lentinan prolongs the survival of patients with advanced gastric cancer, as compared to chemotherapy alone. In addition, trastuzumab, an antibody against HER2/neu growth factor receptor, has been used for the treatment of gastric cancer in combination with cytotoxic chemotherapeutic agents. Lentinan may exert a synergistic action with anti-cancer monoclonal antibodies to activate complement systems through the mechanism of antibody-dependent cellular cytotoxicity and complement dependent cytotoxicity. Because a better understanding of its biological activities should enable us to use lentinan more efficiently in the treatment of gastric cancer, immunological effects provided by β-glucans, a possible mode of action of lentinan, and its clinical application including future potential uses are discussed in the present review.

## INTRODUCTION

Gastric cancer is the second most common cause of cancer-related death in the world [1, 2]. Although both incidence and mortality of this disease have decreased in developed countries, it is a significant problem in global health terms. In particular, unresectable advanced/recurrent gastric cancer remains an incurable disease [3]. Chemotherapy has been used in an attempt to both control cancer-related symptoms and improve survival [3-5]. Despite recent advances in chemotherapy for gastric cancer, the outcomes of anticancer therapy remain unsatisfactory, especially in terms of survival. Thus, further improvement of therapies for gastric cancer is necessary. 

The medicinal qualities of mushrooms have been known for thousands of years and their consumption is an old tradition mainly in China and Japan [6, 7]. Although a number of fungal components have been implicated in these properties, β-glucans were actually identified as biologically active constituents [6, 8]. β-glucans are major cell wall structural components in fungi, yeast, certain bacteria, and cereal plants such as oats and barley [9, 10]. These polysaccharides are well known to be biological response modifiers (BRMs) which stimulate the immune system through activation of various immune cells including macrophages, dendritic cells, neutrophils, natural killer (NK) cells, and lymphocytes. BRMs have been used for cancer therapy in combination with cytotoxic-chemotherapeutic agents [11]. There are several reports describing *in vivo* administration of β-glucans as potentiating the host response against tumor development [12, 13]. In Japan, two types of β-glucans, krestin and lentinan, are licensed as drugs for gastric cancer treatment. Krestin, a protein-bound polysaccharide K (PSK) containing β-(1, 3)-glucan, is derived from *Coriolus versicolor.* This agent has been used clinically in postoperative treatment of resectable gastric cancer [14-16]. However, PSK is not a chemically pure β-glucan and the underlying mechanism is thus rather difficult to elucidate. 

On the other hand, lentinan is purified β-glucan from *Shiitake* mushrooms [17, 18] and has been used in combination with oral fluoropyrimidines for treating gastric cancer in both adjuvant settings and far advanced tumor stages [19, 20]. In this review, we discuss the potential role and future uses of lentinan in the treatment of gastric cancer.

## EFFECTS OF β-GLUCAN ON THE IMMUNE SYSTEM

**Fig. (1):**
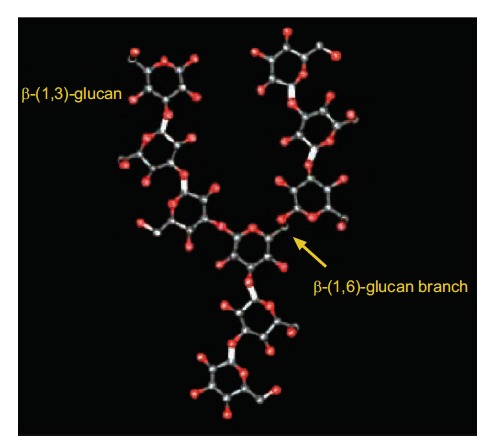
The structure of β-glucans [34]. β-glucans from fungi consist of a backbone of β-(1, 3)-glucan with various degrees of β-(1, 6) glucan branching.

**Fig. (2):**
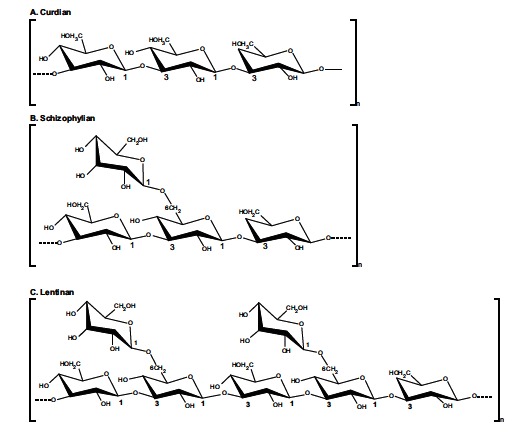
Examples of structures of microbial β-glucans showing the branching patterns of their repeating units [6].

β-glucans from fungi constitute a heterogeneous group of glucose polymers, consisting of a backbone of β-(1, 3)–linked β-D-glucopyranosyl units with β-(1, 6) linked side chains of varying distributions and lengths (Fig. **1**). As β-glucans are not found in animals, they stimulate the immune system and induce innate immune responses, which protect us from attack by pathogenic microbes [6, 9]. The immunomodulatory effects of β-glucans are known to be inconsistent and variable, probably due to differences in the degree of branching, polymer length, and tertiary structures among β-glucans (Fig. **2**). Certain glucans, including zymosan and lentinan appear to efficiently activate phagocytes [21]. Whereas neutrophils are effective against pyogenic bacteria, NK cells circulate in blood to lyse cancer and virus-infected cells. In addition, β-glucans stimulate macrophages to produce cytokines, local immunomodulators, and these in turn activate adaptive immunity against foreign antigens, which involves both B and T cells. B cells produce antibodies to mediate humoral immunity, whereas T cells induce cell-mediated immunity. The adaptive immune response also involves dendritic cells (DCs) derived from monocytes, and these present antigens to T cells for activation of immune responses. There are several reports indicating that DCs are functionally defective in tumor-bearing host [22, 23]. β-glucans were reported to enhance the antigen presenting function of DCs [24], thereby inducing tumor-specific cytotoxic T cells. 

In addition, when the constant region (Fc) of an immunoglobulin interacts with receptors for the Fc domain of IgG (Fc gamma R) on leucocytes, a variety of biological responses are triggered; phagocytosis, enhancement of antigen presentation, release of inflammatory mediators, and antibody-dependent cellular cytotoxicity (ADCC) [25, 26]. Fc gamma R (FcR) provides a critical link between specific humoral responses and cellular immunity. β-glucans were reported to enhance the expression of FcR [27] and the activation of complements [28, 29]. Therefore, β-glucans function actively in cooperation with anti-tumor monoclonal antibodies (mAbs) used in cancer treatment [30, 31].

## POSSIBLE MECHANISMS OF ACTION OF LENTINAN

**Fig. (3):**
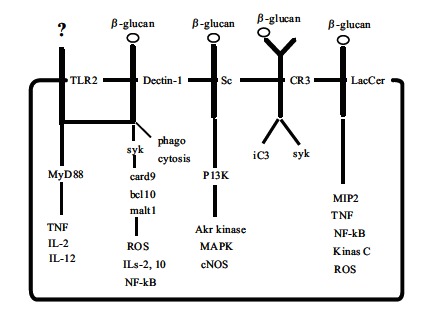
Possible fungal β-glucan mediated signal pathways [6].

β-glucans are recognized *via *a number of cell surface receptors by the immune system as non-self molecules, inducing both innate and adaptive immune responses [6,  21]. Several receptors have been identified in humans, and these include Dectin-1, the toll-like receptor (TLR), complement receptor type 3 (CR3), scavenger receptors, and lactosylceramide (LacCer)　 (Fig. **3**). Dectin-1, the C-type lectin family of receptors, is commonly expressed in macrophages, neutrophils, DCs, and some T-cells, but not in NK cells [32, 33]. Binding of Dectin-1 with β-glucans activates several signaling pathways to promote innate immune responses such as the induction of inflammatory cytokines, activation of phagocytosis, and reactive oxygen species (ROS) production [6, 34]. First, it might act synergistically with TLR to produce various cytokines such as interleukin (IL)-2 and IL-12 through activation of myeloid differentiation primary response gene 88 (MyD88) [35]. Another signaling pathway is mediated by spleen tyrosine kinase (Syk) [36, 37], which in turn activates the caspase recruitment domain 9-bcl10-malt1 complex (CARD9). This complex mediates the induction of NF-kβ [38], which also leads to productions of cytokines such as TNF-α and IL-12. In addition, Dectin-1 can trigger cellular responses to β-glucans independently of the TLRs, including phagocytosis and oxidative burst [33]. The CR3 receptor, leukocyte β2-integrin, consists of CD11b and CD18 domains and is expressed mainly on neutrophils, monocytes, and NK cells, but not macrophages [39, 40]. CR3 functions as an adhesion molecule and there is now evidence that it also activates Syk [41]. NK cells have no Dectin-1 receptor, so CR3 may be the major receptor for NK cells. CR3 on monocytes, granulocytes (neutrophils and eosinophils), and NK cells mediates adherence to inactivated C3b (iC3b)-opsonized targets and involves complement dependent cytotoxicity (CDC) [33]. However, CR3 can be triggered by iC3b deposited onto yeast or fungal cell walls [42], but not cancer cells [43]. This is because the activation of CR3 for CDC requires its dual ligation to both iC3b and cell wall β-glucan [44]. By using β-glucan in combination with anti-tumor mAbs activating complements, the coat of iC3b on tumor cells triggers CR3 dependent cytotoxicity by monocytes, granulocytes, and NK cells [45]. Without β-glucan, anti-tumor mAbs such as trastuzumab and rituximab have limited effector mechanisms including complement-mediated cell lysis and ADCC [46, 47]. The β-glucan primed cells, such as granulocytes and NK cells, then specifically recognize these complement-antibody complexes and kill the coated tumor cells more efficiently. Scavenger receptors located in myeloid and endothelial cells recognize a range of foreign cells, low density lipoprotein, and high-density lipoprotein [48, 49]. Signaling pathways are mediated by phosphatidylinositol-3 kinase (PI3K), leading to stimulation of Akt kinase, MAPK, and an endothelial nitric oxide synthase [50]. LacCer located in neutrophil and endothelial cells is a glycolipid containing a hydrophobic ceramide lipid and a hydrophilic sugar moiety [51]. It has been suggested that the interaction of β-glucan with this receptor can induce macrophage inflammatory protein-2 [52], the activation of NFκB [53], and neutrophil oxidative burst [54]. However, the mechanisms underlying these activities are unknown. 

**Fig. (4):**
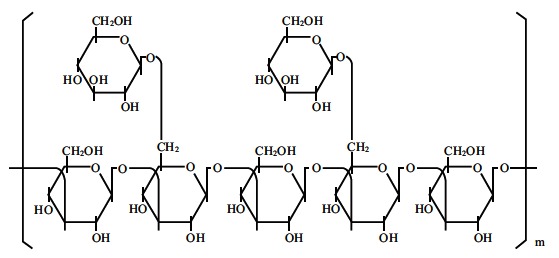
The structure of lentinan.

Lentinan purified from *Shiitake* mushrooms has two β-(1, 6) side chains every five β-(1, 3)–linked backbone residues (Fig. **4**). While the association between lentinan and scavenger receptors and LacCer has not yet been clarified, this immunomodulator is suspected to bind to human leukocytes through both complement receptors, CR1 (CD35) and CR3 (CD11b) as well as Dectin-1 (personal communication: Suga Y, *et al*). In cancer patients, it is well known that DCs are functionally defective [22, 23] and T-cell function as well as NK activity are also down-regulated [55, 56]. Mushiake *et al* described lentinan as activating DC function by increasing the number of tumor-infiltrating CD86+ cells in cancer-bearing mice [24]. The administration of lentinan was reported to stimulate the generation of both killer T cells and NK cells [12, 57, 58] and then restore the ratio of killer/suppressor T cells [59]. Lentinan up-regulated NK cell-mediated killing of tumor cells [60, 61] partly because of increased FcR expression [27], which might be involved in ADCC augmentation. The addition of lentinan activated either the classical or the alternative complement pathway [62] and eventually enhanced CDC and complement-dependent cell-mediated cytotoxicity *via *CR3. Taken together, these observations suggest that immunotherapy using lentinan may have synergistic effects with anti-cancer mAbs [63, 64]. 

**Fig. (5):**
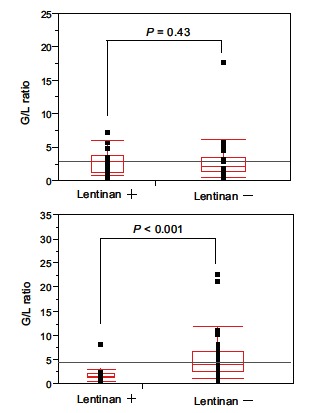
Comparison of the granulocyte/lymphocyte ratio between patients who received (Lentinan+) and those who did not (Lentinan-) using the x^2^ test [73]. **A**: Before therapy. **B**: At either 1 year after initiation of the S-1 based chemotherapy or 1 mo prior to death (cases in which the survival time was < 1 year after starting chemotherapy). *P* values of less than 0.05 were considered statistically significant. G/L ratio: Granulocyte/lymphocyte ratio.

The activation of macrophages and monocytes by binding of lentinan to specific receptors induces IL-12 production [65], although the details of downstream signaling have not been fully elucidated. At the same time, lentinan decreases serum levels of IL-6 and PGE2 in patients with digestive tract cancer [66] and might prevent the Th2-dominant condition. As a result, lentinan induces Th1 polarization and improves the balance between Th1 and Th2. It has been noted that with the progression of cancer, the proportion of granulocytes increases in peripheral blood [15, 67]. Granulocytes reportedly suppress the antitumor activities of lymphocytes and lymphocyte-activated tumor cell killing [68, 69], such that the increased numbers of granulocytes promote tumor growth by antagonizing tumor-suppressing lymphocytes. The increase in granulocytes may be based on the G-CSF increment in cancer patients and the ratio of granulocytes/lymphocytes (G/L ratio) becoming higher in the advanced stage, as compared to the early stage [70, 71]. The G/L ratio was previously reported to correlate with prognosis in gastric cancer patients [72]. The administration of lentinan was found to decrease the serum G-CSF levels in cancer patients [59], which might eventually decrease the G/L ratio. Because the G/L ratio can easily be determined, even in a retrospective analysis, this ratio was chronologically determined as a parameter of its immunological effects in both a group of patients receiving chemotherapy alone and another group given chemotherapy in combination with lentinan [73]. At the start of chemotherapy, the G/L ratio was almost the same with or without lentinan treatment (Fig. **5A**). However, at either 1 year after initiation of chemotherapy or 1 month before death (in cases in which the survival time was less than 1 year after starting chemotherapy), the G/L ratio of patients receiving lentinan was maintained at or below 2, which is significantly lower than those of patients who received chemotherapy alone (*P* < 0.001) (Fig. **5B**). 

## CLINICAL APPLICATION OF LENTINAN IN THE TREATMENT OF GASTRIC CANCER

**Fig. (6):**
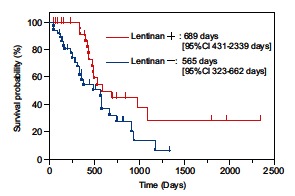
Kaplan-Meier curves of overall survival [73]. Lentinan+: The group that received chemo-immunotherapy with lentinan. Lentinan-: The group that received S-1 based chemotherapy alone. OS: overall survival.

The clinical efficacy of lentinan has been reported in terms of survival in patients with unresectable and recurrent gastric cancer receiving an oral fluoropyrimidine (tegafur) [20]. A meta-analysis conducted by Oba *et al* showed that the addition of lentinan to chemotherapy prolonged the survival of patients with advanced gastric cancer as compared to chemotherapy alone [19]. Although the difference in median overall survival (OS) was statistically significant in this meta-analysis between patients with and without administration lentinan, the increased survivals (139 days versus 114 days; *P* = 0.011) were rather short, compared to those in a recent clinical study [74, 75]. This is explained by the fact that all 5 clinical studies used in this analysis were performed in the 1980s. Oral fluoropyrimidine, S-1, has since been newly combined with 2 modulating substances: gimeracil to inhibit dihydropyrimidine dehydrogenase and potassium oxonate to reduce gastrointestinal toxicities [76]. The anti-tumor effects of fluoropyrimidine are enhanced through biochemical modulation of folate metabolism modified by cisplatin [77], and combination therapy using S-1 and cisplatin reportedly achieves higher response rates than S-1 monotherapy [74, 75, 78]. In addition, taxane derivatives such as docetaxel and paclitaxel have a unique mechanism of action that differs from those of fluoropyrimidines and platinum compounds [79, 80], so that these compounds can be combined with either S-1 or S-1 plus cisplatin for the treatment of advanced gastric cancer [81-84]. As a result, in Japan, these S-1-based regimens are now widely used for the treatment of unresectable or recurrent gastric cancer. However, disease progression is still observed in some patients receiving S-1-based chemotherapy [75, 84, 85] and further improvement of chemotherapy is warranted. With the aim of evaluating the effect of chemo-immunotherapy using lentinan, 78 patients with unresectable/recurrent gastric cancer receiving S-1-based chemotherapy were retrospectively examined and their OS were then compared between treatments with versus without lentinan [73]. S-1-based chemotherapy was continued as long as possible. The median OS was significantly longer in the group that received chemo-immunotherapy with lentinan than in the chemotherapy alone group (689 days [95% CI, 467–2324 days]) versus 565 days [95% CI, 323–662 days]), *P* = 0.0406) (Fig. **6**). One-, two-, and five-year survival rates were better in the group that received lentinan than in the group that received chemotherapy alone, (91.3% versus 59.4%, 45.7% versus 32.7%, 10.0% versus 0%, respectively). These data on S-1-based chemotherapy support the results of a meta-analysis which showed that the addition of lentinan to chemotherapy prolonged the survival of patients with advanced gastric cancer, as compared to chemotherapy alone. 

**Fig. (7):**
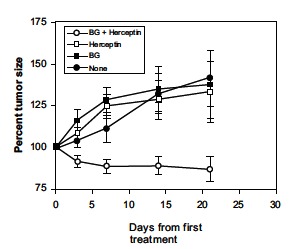
Synergy of β-glucan with anti-HER2 antibody (Herceptin) against human breast carcinoma BT474 xenografts in nude mice [64]. In contrast to controls (solid circles), Herceptin (open squares), or β-glucan controls (BG: solid squares), the combination of Herceptin and β-glucan (open circles) significantly suppressed tumor growth (*P* = 0.002).

Furthermore, recent advances have led to the development of targeted therapies that specifically inhibit the growth of cancer cells [26, 30, 86]. The human epidermal growth factor receptor 2 (HER2) is a member of the ErbB family that plays an important role in promoting oncogenic transformation and tumor growth [87, 88]. Approximately 20-30% of all breast cancers overexpress HER2 [89]. Antibodies against the HER2/neu growth factor receptor prevent the growth of breast carcinoma cells *in vitro* and *in vivo* [90, 91] and target treatment of HER2-positive metastatic breast cancer has been shown to demonstrate favorable efficacy [92, 93]. Trastuzumab, a humanized IgG1 antibody specific for the cellular proto-oncogene HER2/neu, was also approved for the treatment of HER-2 positive gastric cancer [94]. There is evidence supporting a role for trastuzumab in mediating ADCC [95]. The interaction between the Fc domain of trastuzumab and FcR on effecter cells has shown major ADCC involvement and IL-12 enhances cytotoxicity against mAb-coated tumor cells [96, 97]. NK cytotoxicity *via *ADCC is probably one of the mechanisms of action of trastuzumab, but its mode of action includes CDC and complement-dependent cell-mediated cytotoxicity [98, 99]. It has also been reported that platinum compounds exert a synergistic action with trastuzumab in preclinical models of gastric cancer [100]. The objective of a study administering trastuzumab for gastric cancer (ToGA) was to show that addition of this mAb to chemotherapy using capecitabine and cisplatin significantly improved survival in patients with advanced gastric cancer as compared with chemotherapy alone (13.8 months [95% CI,^ 12^-^16^]) versus 11.1 months [95% CI,  10-13]), *P* = 0.0046) [94]. The binding of lentinan to leukocytes could induce IL-12 production and enhance the anti-tumor effects of mAbs through augmented ADCC and complement mediated cytotoxicity *via *CR3 activation. An* in vivo* study clearly demonstrated lentinan to significantly suppress tumor growth in combination with trastuzumab (Fig. **7**) [64]. Considering these properties of lentinan, its synergistic action with targeting cancer therapy might be expected when this immunomodulator is used in combination with trastuzumab plus cytotoxic chemotherapeutic agents for patients with HER-2 positive gastric cancer. 

## CONCLUSION

Chemo-immunotherapy in combination with S-1-based chemotherapy and lentinan might be among the potential candidates for standard treatment of patients with unresectable or recurrent advanced gastric cancer. In Japan, a phase III study comparing therapy using S-1/lentinan with S-1 alone is now under-way. Moreover, as targeted therapy has been applied for gastric cancer, the role of lentinan as an enhancer of the effect of mAb therapy should be examined in clinical settings.
